# LILRB4 regulates multiple myeloma development through STAT3-PFKFB1 pathway

**DOI:** 10.1038/s41419-024-06883-4

**Published:** 2024-07-18

**Authors:** Li Xie, Chiqi Chen, Tinghua Zhang, Wenqian Yang, Denghao Zheng, Liyuan Cao, Jin Yuan, Yilu Xu, Yaping Zhang, Ligen Liu, Aibin Liang, Zhuo Yu, Junke Zheng

**Affiliations:** 1 Hongqiao International Institute of Medicine, Shanghai Tongren Hospital, Key Laboratory of Cell Differentiation and Apoptosis of Chinese Ministry of Education, Faculty of Basic Medicine, Shanghai Jiao Tong University School of Medicine, Shanghai, 200025 China; 2 Academy of Integrative Medicine, Shanghai University of Traditional Chinese Medicine, Shanghai, 201203 China; 3 Department of Hematology, Shanghai Tongji Hospital, Shanghai Tongji University School of Medicine, Shanghai, 200065 China; 4 Shanghai Key Laboratory of Reproductive Medicine, Shanghai Jiao Tong University School of Medicine, Shanghai, China

**Keywords:** Cancer metabolism, Myeloma

## Abstract

Although multiple myeloma (MM) responds well to immunotherapeutic treatment, certain portions of MM are still unresponsive or relapse after immunotherapy. Other immune molecules are needed for the immunotherapy of MM. Here, we revealed that leukocyte immunoglobulin-like receptor B4 (LILRB4) was highly expressed in multiple myeloma cell lines and patient samples and that the expression of LILRB4 was adversely correlated with the overall survival of MM patients. Knockdown of LILRB4 efficiently delayed the growth of MM cells both in vitro and in vivo. Mechanistically, IKZF1 transactivated LILRB4 expression to trigger the downstream of STAT3-PFKFB1 pathways to support MM cell proliferation. Blockade of LILRB4 signaling by blocking antibodies can effectively inhibit MM progression. Our data show that targeting LILRB4 is potentially an additional therapeutic strategy for the immunotherapeutic treatment of MM.

## Introduction

Multiple myeloma (MM) is a hematopoietic malignancy with increased monoclonal plasma cells in bone marrow, eventually leading to severe infiltration and dysfunction in multiple organs, including renal failure [1], anemia [2], and bone lesions [3]. Multiple myeloma typically begins as a monoclonal gammopathy of undetermined significance (MGUS) [3, 4]. Thus, a precancerous condition in which asymptomatic people have full or partial antibodies called monoclonal protein (M protein) in their blood [5]. Abundant M proteins produced by abnormal plasma cells can eventually accumulate in kidneys [6] and impair kidney function. The marked increase in abnormal plasma cells in the bone marrow can also lead to bone destruction [7], hypercalcemia [8], anemia, infection [9] and neurological symptoms [10]. Multiple myeloma (MM) is currently considered as an incurable malignant disorder and urgently required for the novel and effective therapeutic targets. B cell maturation antigen (BCMA) is an effective target for MM due to its highly selective expression in malignant plasma cells (PCs), which shows effective clinical response in patients with relapsed and refractory MM with the treatment of BCMA antibodies as well as its related chimeric antigen receptor (CAR)-T cells. Although many therapeutic ways, including immunomodulatory drugs (IMiDs) [11], proteasome inhibitors (PIs) [12] and corticosteroids [13], show significant effect in delay of MM development, but the immune evasion and drug resistance still frequently occur after treatment. Ikaros family zinc finger protein 1 (IKZF1) is a transcription factor with multiple roles in hematopoiesis and also promotes the MM development [14, 15]. Interestingly, the inhibition of IKZF1 by Lenalidomide leads to the cell death of myeloma cell death through the Cereblon (CRBN)-dependent ubiquitylation [16], indicating it may serve as a potential target in developing the strategies for MM treatment.

LILRB4 is a member of the LILRB family and is mainly expressed on several myeloid cells, including DCs [17], monocytes [18], and macrophages [19]. Interestingly, it has also been reported that many tumor cells, such as solid tumors and hematological tumors, highly express LILRB4 [20, 21]. Our previous studies have shown that LILRB4 is specifically expressed on monocytic AML cells and bind to apolipoprotein E (APOE) and to recruit SHP-2 to its intracellular ITIMs, then followed by the activation of the downstream NF-kB signaling pathway to promote leukemia cell infiltration and T-cell inhibition [20]. However, whether LILRB4 is expressed on MM cells and its function in MM development remain unknown.

It is well known that cancer cells usually utilize glycolysis as the main energy source [22]. Previous evidence has suggested that MM cells have unique metabolic profiles, which are tightly connected with their cell fate determinations [23–26]. For example, Rizzieri showed that MM cells have increased glycolysis and oxidative phosphorylation (OXPHOS) levels to sustain their proliferation capacities, accompanied by recurrent genetic aberrations in cells [27]. PGC-1α controls OXPHOS levels to enhance MM progression [28]. Therefore, it seems that the metabolic mechanism, especially glucose metabolism, in MM remains controversial, and the key regulatory network related to the dynamic metabolic changes in MM cells is unclear.

In the present study, we demonstrated that LILRB4 was highly expressed in MM cells and adversely associated with the overall survival of MM patients. LILRB4 fine-tunes MM cell proliferation and glycolysis levels through the STAT3-PFKFB1 pathway. IKZF1 can directly transactivate LILRB4 to promote MM development. Blocking LILRB4 with a blocking antibody can efficiently suppress MM cell growth. Our data indicate that targeting LILRB4 may be another potential immunotherapeutic method for MM treatment.

## Results

### LILRB4 is highly expressed on multiple myeloma cells

**Fig. 1:**
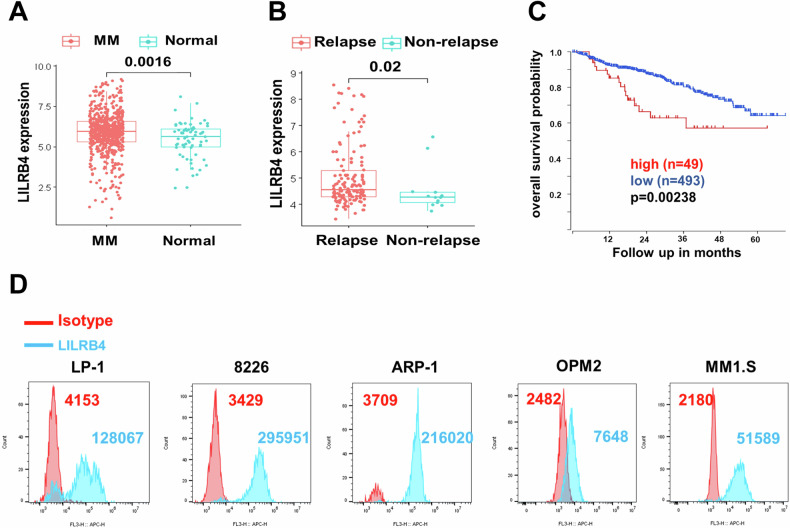
LILRB4 is highly expressed in multiple myeloma cells. **A** LILRB4 expression was measured in bone marrow cells from healthy donors and multiple myeloma patients (n = 682) from the TCGA database GSE118985. **B** LILRB4 expression levels were compared in bone marrow cells from relapsed and nonrelapsed MM patients (n = 157) from the TCGA database GSE83503. **C** Analysis of correlations between LILRB4 mRNA levels and the overall survival of MM patients (n = 542) from the TCGA database GSE2658 (Low, n = 493; High, n = 49). **D** LILRB4 expression levels were quantified by flow cytometric analysis in LP-1, 8226, ARP-1, OPM2 and MM1.S cells.

A previous study showed that LILRB4 was highly expressed in AML cells according to TCGA databases [20]. However, few data demonstrate the expression pattern of LILRB4 in MM cells and the potential connection between its expression levels and the overall survival of patients. We first analyzed the expression of LILRB4 in MM using 682 MM patient samples from the GEO database (GSE118985). We found that LILRB4 levels were much higher in MM patients than in healthy donors (Fig. 1A). We also found that LILRB4 was highly expressed on MM cells from relapsed patients (Fig. 1B). Consistently, the expression of LILRB4 was adversely correlated with the overall survival of MM patients (Fig. 1C). We further examined LILRB4 levels in several MM cell lines (LP-1, 8226, ARP-1, OPM2 and MM1.S), and revealed that LILRB4 was highly expressed in MM cells compared to the normal B cells (Fig. 1D and sFig. 1A–C), indicating that LILRB4 may play a critical role in MM development.

### LILRB4 supports MM cell proliferation in vitro

**Fig. 2:**
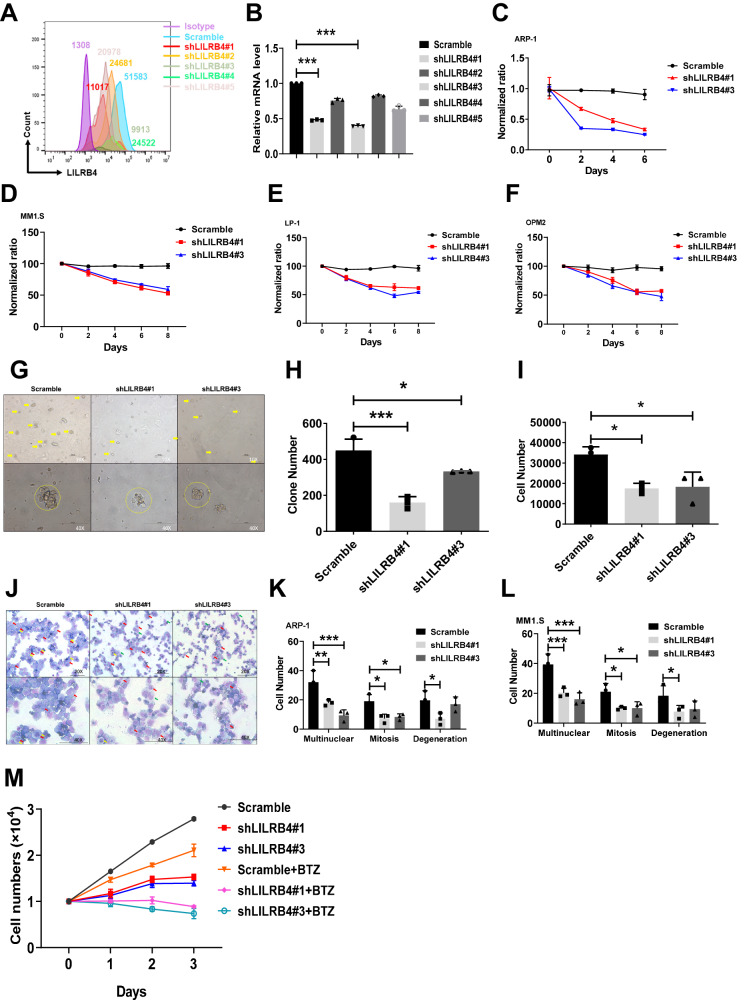
LILRB4 supports MM cell proliferation in vitro. **A** The knockdown efficiency of shRNAs targeting human LILRB4 were determined in ARP-1 cells by flowcytometric analysis. **B** Relative mRNA levels of LILRB4 were measured in ARP-1 cells upon LILRB4 knockdown by shRNAs (shLILRB4#1, 2#, 3#, 4#, and #5, n = 3). **C**–**F** The numbers of MM cells were counted on the indicated days upon LILRB4 knockdown by shRNAs (shLILRB4#1 and #3) and a scrambled control. Four human MM cell lines were used for the indicated experiments (ARP-1, MM1.S, LP-1 and OPM2) (n = 3). Representative images (**G**) of colonies derived from scrambled or LILRB4-knockdown (shLILRB4#1 and #3) ARP-1 cells. Quantitative analysis of colony numbers (**H**) and total cell numbers (**I**) derived from scrambled or LILRB4-knockdown cells in **F** (n = 3). Representative images of Wright-Giemsa staining of scrambled or LILRB4-knockdown APR-1 cells. Quantitative data in **J** are shown (**K**), and a total of 25-70 ARP-1 cells were counted (n = 3). Red, yellow, and green arrows are indicated multinuclear cells, mitosis cells and degeneration cells. **L** Quantitative data of Wright-Giemsa staining in scrambled or LILRB4-knockdown MM1.S cells. A total of 37-80 MM1.S cells were counted (n = 3). **M** The LILRB4-knockdown (shLILRB4#1 and #3) ARP-1 cells and scrambled ones were treated with or without bortezomib (BTZ) and the cell numbers in each group were counted at indicated days upon treatment (n = 3). Data was represented as the mean ± SEM. One-way ANOVA with Tukey’s multiple comparison test (**A**, **H**, **I**) and two-way ANOVA with Sidak’s multiple comparison test (**B**, **C**, **D**, **E**, **F**, **K**, **L**) were used for the comparison of statistical significance. *p < 0.05; **p < 0.01; ***p < 0.001.

To evaluate LILRB4 function in MM cells, we constructed several short hairpin RNAs (shRNAs) and evaluated their knockdown efficiency in ARP-1 cells by FACS and qRT‒PCR (Fig. 2A, B). Knockdown of LILRB4 in MM cells, including ARP-1 and MM1.S cells, LP-1 and OPM2, resulted in a significant decrease in cell proliferation in vitro (Fig. 2C–F). An in vitro colony formation assay also revealed that knockdown of LILRB4 in ARP-1 cells resulted in decreased colony numbers and total derived cell numbers (Fig. 2G–I). Wright–Giemsa staining-based morphological analysis demonstrated that LILRB4-knockdown ARP-1 and MM1.S cells exhibited a decreased cytoplasm-to-nucleus ratio and an increased percentage of abnormal nuclear segmentation or bending (Fig. 2J–L). Furthermore, LILRB4-knockdown ARP-1, MM1.S, and LP-1 cells had much higher early and late apoptotic cells than scrambled cells as determined by annexin V/propidium iodide staining (sFig 2A–F). Additionally, we treated these cells with Bortezomib (BTZ), which belongs to one type of proteasome inhibitors and can efficiently suppress the proteasome’s enzyme activity to lead to cell cycle arrest and apoptosis of multiple myeloma cells. We found that knockdown of the expression of LILRB4 could significantly increase the chemosensitivity of multiple myeloma cells, as evidenced by a synergistically effect in suppression of cell proliferation (Fig. 2M). These results indicate that LILRB4 serves as a potent oncogene to promote MM cell proliferation in vitro.

### LILRB4 promotes MM development in vivo

**Fig. 3:**
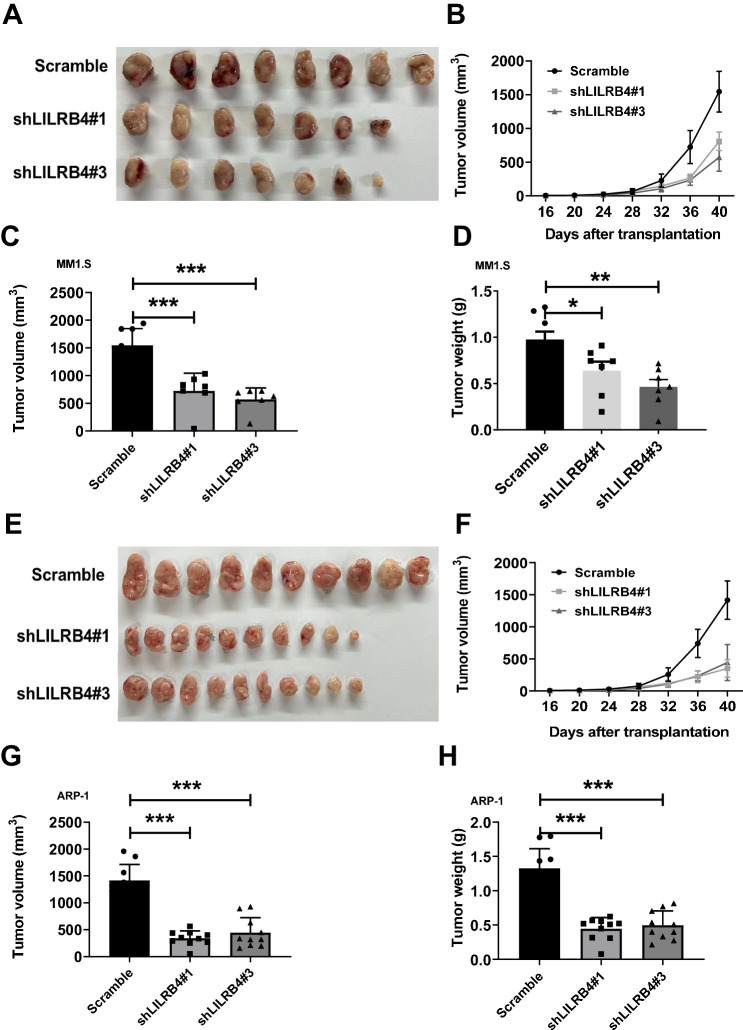
LILRB4 promotes MM development in vivo. **A**–**D** Representative images of tumors in NOG-SCID mice subcutaneously injected with 2 × 10^6^ MM1.S cells. Quantitative data of tumor growth, weights and volumes in Panel A are shown (**B**–**D**) (n = 7-8). **E**–**H** Representative images of tumors in NOG-SCID mice subcutaneously injected with 2 × 10^6^ ARP-1 cells. Quantitative data of tumor growth, weights and volumes in **E** are shown (**F**–**H**) (n = 10). Data are represented as the mean ± SEM. One-way ANOVA with Tukey’s multiple comparison test (**B**, **C**, **D**, **F**, **G**, **H**) was used for the comparison of statistical significance. *p < 0.05; **p < 0.01; ***p < 0.001.

To further confirm the function of LILRB4 in MM development in vivo, 2 × 10^6^ LILRB4-shRNA-infected MM1.S cells were subcutaneously injected into NOG-SCID mice. The mice were sacrificed, and the sizes of the tumors were photographed and measured five weeks later (Fig. 3A). Strikingly, the tumor sizes or weights derived from mice with LILRB4-knockdown MM cells were much smaller or less than those derived from mice with the scrambled cells (Fig. 3B–D). Similarly, LILRB4-knockdown APR-1 cells grew much slower in vivo than scrambled cells (Fig. 3E–H). These findings suggest that LILRB4 promotes the proliferation of MM cells in vivo.

### LILRB4-PFKFB1 pathways sustain MM cell tumorigenic activities

**Fig. 4:**
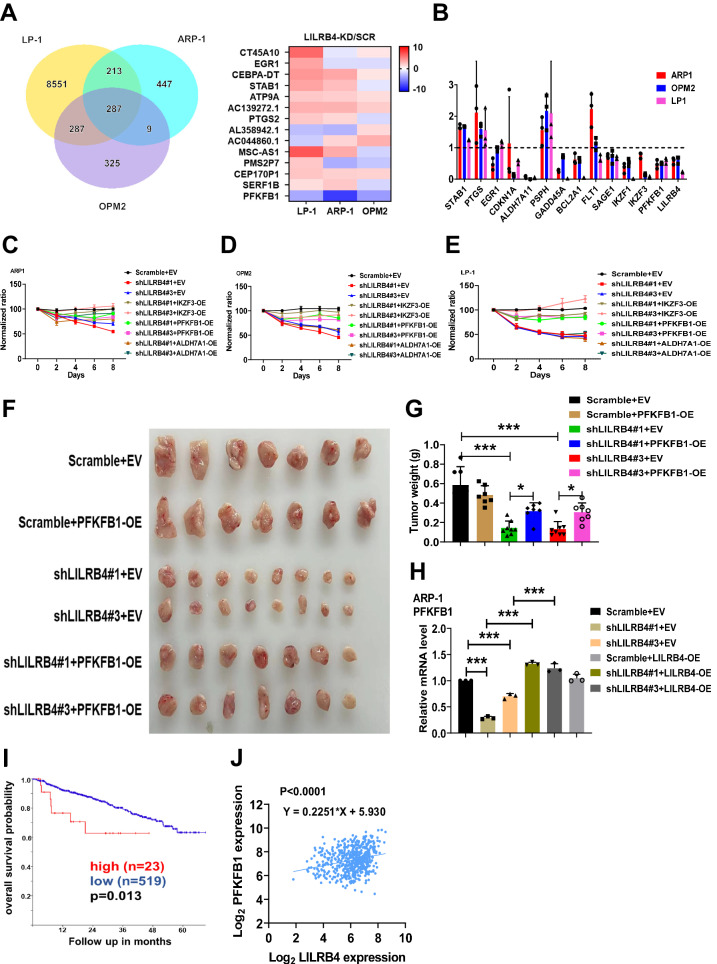
The LILRB4-PFKFB1 pathways sustain MM cell tumorigenic activities. **A** RNA-sequencing analysis was performed in scrambled or LILRB4-knockdown APR-1, LP-1 and OPM2 cells. **B** Quantitative RT‒PCR analysis of the expression levels of candidate genes in scrambled or LILRB4-knockdown APR-1, OPM2 and LP-1 cells (n = 3). **C**–**E** The numbers of MM cells were counted on the indicated days in scrambled control cells and LILRB4-knockdown (shLILRB4#1 and #3) MM cells with IKZF3-, PFKFB1- or ALDH7A1-overexpression. Three human MM cell lines, ARP-1 (**C**), OPM2 (**D**) and LP-1 (E), were used for the indicated experiments (n = 3). **F**, **G** Representative images (**F**) of tumors in NOG-SCID mice subcutaneously injected with 2 × 10^6^ Scrambled (Scrambled+EV) or LILRB4-knockdown (shLILRB4#1 + EV and #3 + EV) MM cells and Scrambled or LILRB4-knockdown MM cells with PFKFB1 overexpression (Scrambled+PFKFB1-OE, shLILRB4#1 + PFKFB1-OE, shLILRB4#3 + PFKFB1-OE) ARP-1 cells. Quantitative data of tumor weights in **F** are shown (**G**) (n = 7–8). **H** Quantitative RT-PCR analysis of the expression level of PFKFB1 in Scramble+EV, shLILRB4#1 + EV, shLILRB4#1 + LILRB4-OE, shLILRB4#3 + EV and shLILRB4#3 + LILRB4-OE ARP-1 cells (n = 3). **I** Analysis of correlations between PFKFB1 mRNA levels and the overall survival of MM patients (n = 542) from the GEO database. (Low, n = 519; High, n = 23). **J** Analysis of correlations between PFKFB1 and LILRB4 mRNA levels in MM patients (n = 559) from the GEO database. Data are represented as the mean ± SEM. One-way ANOVA with Tukey’s multiple comparison test (**G**, **H**) and two-way ANOVA with Sidak’s multiple comparison test (**B**, **C**, **D**, **E**) were used for the comparison of statistical significance. *p < 0.05; **p < 0.01; ***p < 0.001.

For better understanding of the molecular mechanisms that regulate the proliferation capability and tumorigenic activity of MM cells, we performed RNA-sequencing analyses using LILRB4-knockdown and scrambled MM cells (ARP-1, OPM-2 and LP-1 cells) and demonstrated that approximately 14 genes were markedly decreased upon LILRB4 knockdown, including CT45A10, EGR1, CEBPA-DT, STAB1, ATP9A, PTGS2, SERF1B, PFKFB1, PMS2P7, CEP17OP1, and MSC-AS1 (Fig. 4A). Then, quantitative RT‒PCR analysis was performed to examine the mRNA levels of candidate genes, including ALDH7A11, GADD45A, BCL2A1, IKZF2, IKZF3, and PFKFB1, which were significantly reduced in all three tested LILRB4-knockdown MM cell lines (ARP1, OPM2 and LP1 cells) (Fig. 4B). Interestingly, we noticed that PFKFB1, as a key regulator of glycolysis, was expressed at a higher level in scrambled MM cells than in LILRB4-knockdown cells. PFKFB1 has been reported to serve as the key enzyme for the generation of fructose-2,6-bisphosphate (F-2,6-BP), which is the most potent allosteric activator of phosphofructokinase 1 (PFK1). PFK1 is another pivotal enzyme for glycolytic flux. This evidence suggests that PFKFB1 may be involved in the proliferation and metabolic regulation of MM cells. To evaluate the function of PFKFB1 in tumorigenesis, we constructed a PFKFB1 overexpression vector and infected it in LILRB4-knockdown (shLILRB4#1 and #3) and scrambled MM cells. Overexpression of PFKFB1 in LILRB4-knockdown MM cells resulted in a significant increase in proliferation ability in vitro compared with LILRB4-knockdown MM cells (Fig. 4C). We also detected the function of other candidate targets downstream of the LILRB4, including IKZF3, which could maintain the proliferation ability of MM cells (Fig. 4D, E). Moreover, the recipient mice receiving PFKFB1-overexpressing LILRB4-knockdown ARP-1 cells had enhanced growth capability compared with that of the LILRB4-knockdown ARP-1 mice, as exhibited by the increased tumor sizes and tumor weights (Fig. 4F, G). Alternatively, overexpression of LILRB4 in LILRB4-knockdown ARP-1 cells also increased the mRNA level of PFKFB1 (Fig. 4H). Furthermore, the expression level of PFKFB1 was adversely correlated with the overall survival of MM patients (Fig. 4I) and positively correlated with LILRB4 expression in MM patients (Fig. 4J). This evidence suggests that PFKFB1 can partially rescue the LILRB4 knockdown effect in MM cells and enhance the proliferation of MM cells.

### LILRB4 is critical for the maintenance of the metabolic status of MM cells

**Fig. 5:**
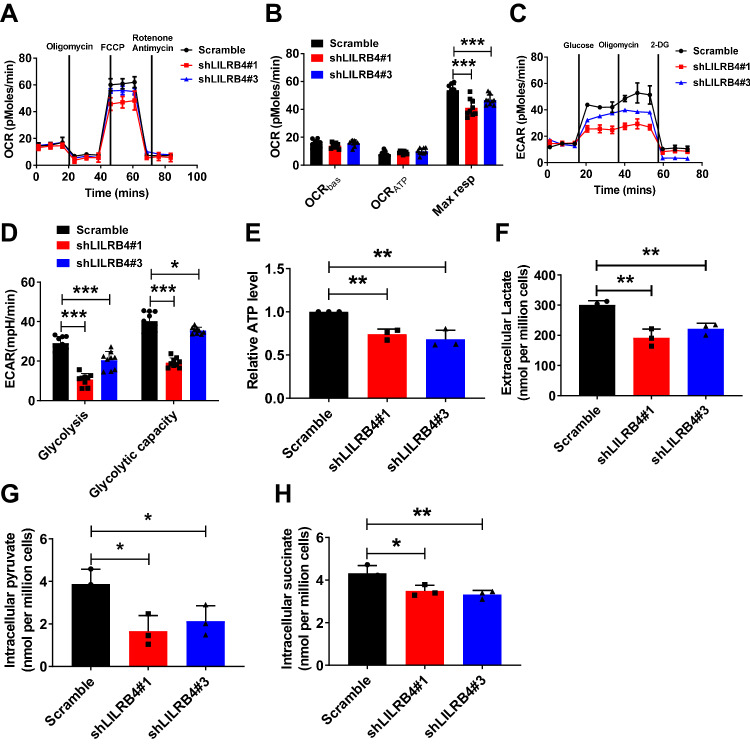
LILRB4 is critical for the maintenance of the metabolic status of MM cells. **A**, **B** Oxygen consumption rates (OCR) were detected in LILRB4-knockdown (shLILRB4#1 and #3) and scrambled control ARP-1 cells using a Seahorse XF96 analyzer (n = 3). OCR_bas_, OCR_ATP_, and max respiration in **A** were analyzed (**B**) (n = 3). **C**, **D** Extracellular acidification rates (ECAR) were measured in LILRB4-knockdown (shLILRB4#1 and #3) and scrambled control ARP-1 cells using a Seahorse XF96 analyzer (n = 3). Glycolysis and glycolysis capacity in **C** were analyzed (**D**) (n = 3). **E** ATP levels were measured in LILRB4-knockdown (shLILRB4#1 and #3) and scrambled control ARP-1 cells (n = 3). **F** Extracellular lactate levels in LILRB4-knockdown (shLILRB4#1 and #3) and scrambled control ARP-1 cells (n = 3). **G**, **H** Intracellular pyruvate and succinate levels in LILRB4-knockdown (shLILRB4#1 and #3) and scrambled control ARP-1 cells. Data are represented as the mean ± SEM. One-way ANOVA with Tukey’s multiple comparison test (**E**, **F**, **G**, **H**) and two-way ANOVA with Sidak’s multiple comparison test (**B**, **D**) were used for the comparison of statistical significance. *p < 0.05; **p < 0.01; ***p < 0.001.

To reveal the molecular mechanisms by which LILRB4 regulates the glucose metabolism of MM cells, several biochemical analyses were performed in LILRB4-knockdown MM cells. Interestingly, the LILRB4-knockdown ARP-1 cells had a slight decrease in the oxygen consumption rate (OCR) (Fig. 5A, B) but had a much lower extracellular acidification rate (ECAR), as evidenced by the decrease in basal glycolysis and glycolytic capability (Fig. 5C, D). Consistently, the levels of adenosine 5′-triphosphate (ATP), lactate, pyruvate and succinate were also decreased in LILRB4 knockdown ARP-1 cells (Fig. 5E–H). This evidence suggests that LILRB4 plays a critical role in sustaining both high levels of glycolysis and certain levels of oxidative phosphorylation in MM cells.

### IKZF1-LILRB4-STAT3 signaling pathways transactivate the expression of PFKFB1

**Fig. 6:**
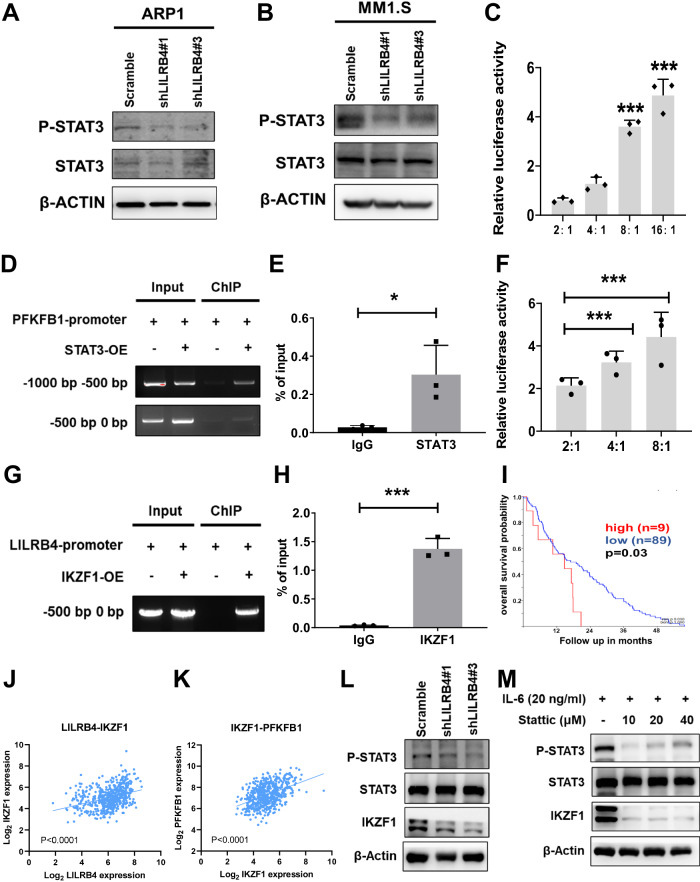
IKZF1-LILRB4-STAT3 signaling pathways transactivate the expression of PFKFB1. **A**, **B** STAT3 and p-STAT3 levels were measured in LILRB4-knockdown (shLILRB4#1 and #3) and scrambled control ARP-1 and MM1.S cells by western blot. (C) PFKFB1 luciferase reporter and different doses of STAT3 were cotransfected into 293T cells, followed by the determination of luciferase activities (n = 3). **D**, **E** ChIP assays were analyzed with 293 T cells transfected with STAT3 overexpression plasmid and empty vector. Input control and amplification of the STAT3 binding sequence of PFKFB1 were determined by semiquantitative PCR. Quantitative data in Panel E are presented as the percentage of input DNA (**E**) (n = 3). **F** LILRB4 luciferase reporter and different doses of IKZF1 were cotransfected into 293T cells, followed by the determination of luciferase activities (n = 3). **G**, **H** ChIP assays were analyzed with 293T cells transfected with the IKZF1 overexpression plasmid and empty vector. Input control and the amplification of the IKZF1 binding sequence of LILRB4 were determined by semiquantitative PCR. Quantitative data in Panel G are presented as the percentage of input DNA (H) (n = 3). **I** Analysis of correlations between IKZF1 mRNA levels and the overall survival of MM patients (n = 98) from the GEO database. (Low, n = 89; High, n = 9). **J** Analysis of correlations between IKZF1 and LILRB4 mRNA levels in MM patients (n = 559) from the GEO database. **K** Analysis of correlations between IKZF1 and PFKFB1 mRNA levels in MM patients (n = 559) from the GEO database. **L** p-STAT3 and IKZF1 levels were measured in LILRB4-knockdown (shLILRB4#1 and #3) and scrambled control ARP-1 cells by western blot. **M** p-STAT3 and IKZF1 levels were measured in Stattic-treated ARP1 cells by western blot. IL-6 was added to enhance STAT3 signaling pathway. Data are represented as the mean ± SEM. Student’s two-tailed unpaired t test (**E**, **H**) and one-way ANOVA with Tukey’s multiple comparison test (**C**, **F**) were used for the comparison of statistical significance. *p < 0.05; **p < 0.01; ***p < 0.001.

To further reveal how LILRB4 fine-tunes the tumorigenesis and metabolic profiles of MM cells, we evaluated several signaling pathways markedly changed in LILRB4-knockdown MM cells according to the RNA-seq data, such as the JAK-STAT signaling pathway and PI3K-AKT signaling pathway. We found that the phosphorylation level of signal transducer and activator of transcription 3 (STAT3) was significantly downregulated in LILRB4-knockdown ARP-1 and MM1.S cells (Fig. 6A, B). STAT3 efficiently transactivated PFKFB1 expression in a dose-dependent manner (Fig. 6C). Consistent with the luciferase assay results, the chromatin immunoprecipitation (ChIP) assay further showed that STAT3 could bind to the promoters of PFKFB1 and transactivate the expression of PFKFB1 (Fig. 6D, E). To understand how the LILRB4 expression level is regulated, we noticed that IKZF1 was downregulated in LILRB4-knockdown MM cells according to the RNA-seq and qRT-PCR data. We further demonstrated that IKZF1 could efficiently transactivate LILRB4 expression in a dose-dependent manner (Fig. 6F). ChIP analysis also showed that IKZF1 could bind to the promoters of LILRB4 and transactivate the expression of LILRB4 (Fig. 6G, H). Consistently, we also found that the expression of IKZF1 was adversely correlated with the overall survival of MM patients (Fig. 6I). The expression of IKZF1 was positively related to the expression of LILRB4 and PFKFB1 in MM patients (Fig. 6J, K). To further evaluate the potential connections between IKZF1 and LILRB4, we examined the protein levels of IKZF1 upon LILRB4 knockdown, which showed that IKZF1 was also markedly decreased (Fig. 6L). Meanwhile, the protein level of IKZF1 was significantly decreased in MM cells upon the treatment with a specific STAT3 inhibitor, Stattic. These results suggest that STAT3 enhance the expression of IKZF1, which further transactivate the expression of LILRB4 in a feedback manner (Fig. 6M). In summary, the IKZF1-LILRB4-STAT3-PFKFB1 pathways may be involved in maintaining the proliferation capability and metabolic profiles of MM cells.

### Blockade of LILRB4 with blocking antibody inhibits MM cell proliferation

**Fig. 7:**
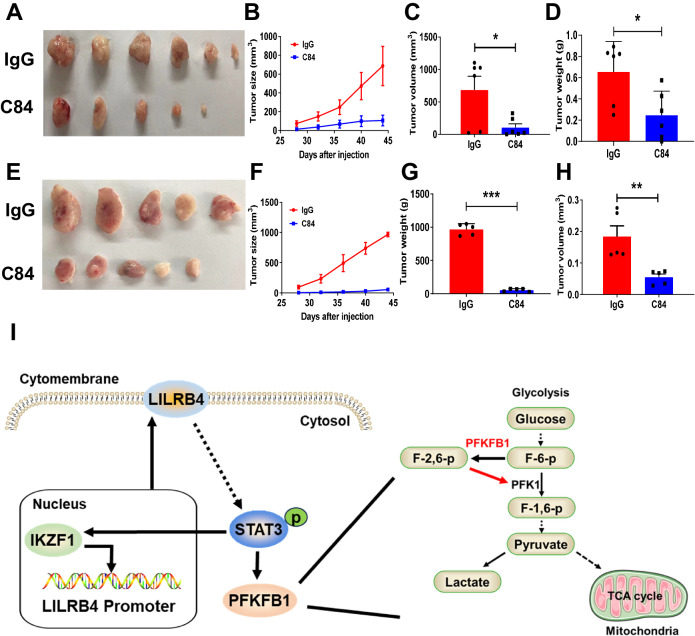
Blockade of LILRB4 with blocking antibodies inhibits MM cell proliferation. **A**–**D** ARP-1 cells (2Х10^6^) were subcutaneously injected into NOG-SCID mice, followed by treatment with IgG or an anti-LILRB4 antibody (C84). Representative images of tumors are shown (**A**). Tumor sizes were measured on the indicated days (**B**). Quantitative data of tumor weights and volumes in Panel A after mice were sacrificed are shown (**C**, **D**) (n = 6). (**E**-**H**) 2Х10^6^ MM1. S cells were injected subcutaneously into NOG-SCID mice, followed by treatment with IgG or anti-LILRB4 antibody (C84). Representative images of tumors are shown (**E**). Tumor sizes were measured on the indicated days (**F**). Quantitative data of tumor weights and volumes in Panel A after mice were sacrificed are shown (**F**–**H**) (n = 5). **I** Working model for LILRB4 function in MM cell proliferation. Data are represented as the mean ± SEM. Student’s two-tailed unpaired t test (**C**, **D**, **G**, **H**) and one-way ANOVA with Tukey’s multiple comparison test (**B**, **F**) were used for the comparison of statistical significance. *p < 0.05; **p < 0.01; ***p < 0.001.

In our previous study, we showed that LILRB4 blockade with a blocking antibody can enhance CD8 T-cell cytotoxicity to suppress AML cell growth and infiltration into different organs. For better understanding of anti-LILRB4 function in MM progression, we subcutaneously implanted ARP-1 and MM1.S cells into NOG-SCID mice and treated them with anti-LILRB4-specific monoclonal antibodies (C84) [20] or IgG control antibodies. We found that LILRB4 blockade effectively decreased ARP-1 growth in vivo, as exhibited by reduced tumor size and volume compared to those in the control group (Fig. 7A–D). Consistently, C84 effectively inhibited the proliferation of MM1.S cells in vivo (Fig. 7E–H). In summary, we herein showed that LILRB4 was highly expressed in both MM cell lines and patient samples. Knockdown of LILRB4 efficiently inhibited the proliferation of MM cells both in vitro and in vivo. The IKZF1-LILRB4-STAT3-PFKFB1 pathways are critical for the maintenance of tumorigenic activities and metabolic status in MM cells (Fig. 7I, working model). Targeting LILRB4 using blocking antibody may serve as another potential way to treat MM.

## Discussion

In our previous study, we showed that LILRB4 serves as a potent immune checkpoint in AML development [20]. However, the function of LILRB4 in MM development remains unclear. We herein extended our studies and revealed that LILRB4 is highly expressed on human MM cells and adversely correlated with the overall survival of MM patients. We showed that LILRB4 sustains the proliferation and metabolic status of MM cells via STAT3-PFKFB1 pathways. Moreover, blocking LILRB4 signaling sufficiently decreases MM development. Although we herein showed that LILRB4 supports MM cell growth in an autonomous manner, whether LILRB4 can suppress T-cell function remains unclear. Since we previously showed that LILRB4 could enhance SHP2-NF-KB-ARG1 pathways to inhibit T-cell activities, we further scrutinized these signaling pathways in the MM model. We are also eager to understand whether certain surface ligands can directly bind to LILRB4 and enhance MM growth.

Immunotherapy has been considered an effective method for MM treatment. Immunomodulatory drugs (IMiDs) have direct antiproliferative and proapoptotic effects in multiple myeloma cells. However, these drugs also exhibit indirect effect in regulating MM cell activities by modulating various immune effector cells, which may reduce the immunosuppressive effects [12, 29]. Interestingly, IKZF1 has been reported to serve as the target of Lenalidomide to sustain the proliferation of MM cells. Lenalidomide effectively enhances the interaction between E3 ligase CRBN and IKZF1, which leads to the accelerated IKZF1 proteasome-dependent degradation and inhibition of MM proliferation [30–32]. Herein, we showed that IKZF1 may enhance LILRB4 expression and promote MM progression via PFKFB1 pathway and blocking with LILRB4 antibody showed notable effect in suppression of MM cell growth, indicating the combined treatment with IKZF1 or LILRB4 inhibition may be a new way for MM treatment. BCMA is one of the immune molecules widely used for CAR-T-based immunotherapeutic treatment [33]. However, approximately 71.4% of patients are unresponsive and relapse after BCMA-CAR-T treatment [34]. One of the main reasons for BCMA antigen escape is heterogeneously expressed BMCA in MM cells, which can lead to targeting of BCMA-high cells and result in the outgrowth of BCMA-low clones [35–37]. Another persistent problem with CAR-T therapy is toxicity, such as cytokine release syndrome (CRS) and neurotoxicity, mediated by proinflammatory cytokines. BCMA is not only a plasma cell or MM cell marker but is also coexpressed on normal B cell lymphocytes; therefore, BCMA CAR T-cell therapy could also result in B-cell aplasia, which leads to increased infection risks [38]. Recently, GPCR5D-CAR-T cells have been reported for combinational treatment with MM patients with no response to BCMA-based immunotherapy [39], which notably improved the efficiency of immunotherapy. However, CAR-T-based treatment is currently expensive and inconvenient. Antibody-based immunotherapies targeting the malignant plasma cell-specific antigens CD38, CD138, BCMA, FcRH5, and GPRC5D have demonstrated a safety profile, with mainly low-grade cytokine release syndrome, cytopenias, and infections [40, 41]. Emerging data report that antibody elotuzumab, are in advanced stages of development and are expected to have a major impact on the management of MM [33, 39, 40]. Therefore, antibody-based immunotherapy may be a potent method for MM treatment, although there is a lack of ideal candidate of immune molecules [39]. The underlying mechanisms for therapeutic resistance are still unclear regarding tumor heterogeneity and antigen escape; toxicity, such as CRS and neurotoxicity, mediated by proinflammatory cytokines, is another persistent problem. MM cell markers, including BCMA and CD38, were also coexpressed on normal B lymphocytes; therefore, anti-BCMA antibody-based immunotherapy could also result in B-cell aplasia, neutropenia, and immunosuppression, leading to increased infection risks. Our data showed that blocking antibodies against LILRB4 can specifically and efficiently delay MM cell growth in vivo, indicating that LILRB4 may be another potent target for immunotherapy. It also suggested that LILRB4-CAR-T-based immunotherapy may be another potential method for MM treatment. More efforts are needed to evaluate the possible immune strategy in MM treatment by targeting LILRB4.

It has been reported that the occurrence of tumors is often accompanied by dramatic metabolic changes, which may be driven by oncogenes and genomic mutations [42, 43]. For example, the initiation of leukemia is often involved in metabolic reprogramming [44, 45]; the mTORC pathway has been shown to induce the expression of PFKFB3 via HIF1 signaling to enhance glycolysis in AML cells [46]; NOX-mediated ROS levels also promote PFKFB3 expression via AMPK pathways to sustain the proliferation and survival of AML cells [47]. Similar to other cancer types, MM cells prefer to utilize glycolysis as the main energy source [24, 48, 49]. Recent studies have shown that the Warburg effect is markedly increased in MM cells and that several key enzymes involved in glycolysis, such as LDHA, GLUT1 and PDK1, are significantly increased during the progression of MM. Specific inhibition or knockdown of LDHA and HIF1A can restore sensitivity to therapeutic agents such as bortezomib and can also inhibit tumor growth induced by altered metabolism [50]. Recent study revealed that the increased expression of phosphofructokinase platelet (PFKP) is tightly connected with metabolic activities to reduce the chemotherapy sensitivity in leukemia initiating cells (LICs) [51]. Another member of the PFK family, PFKL, is also highly expressed in MM cells, which can be transactivated by STAT3 signaling [52]. Consistently, we have shown that the glycolysis rate-limiting enzyme PFKFB1 is highly expressed in MM and is associated with poor prognosis [53]. We herein also showed that PFKFB1 serves as a potential downstream target of LILRB4 to promote MM cell proliferation both in vitro and in vivo and to maintain a high level of glycolysis. To our knowledge, this is the first line of evidence showing that LILRB4 maintains glycolysis levels in cancer cells, although the signaling pathway of LILRB4 involved in metabolism remains unclear. We also noticed that knockdown of LILRB4 led to a slight decrease in oxidative phosphorylation, indicating that this metabolic pathway is needed for MM cell growth, although more ROS may be generated during oxidative phosphorylation. It’s demonstrated that cancer cells (such as MM) may adopt several different metabolic pathways to satisfy energy demands. Although we showed that STAT3 transactivates the expression of PFKFB1 to sustain the tumorigenic activities and metabolic status of MM cells, the detailed mechanism related to how LILRB4 affects glucose metabolism in MM awaits further investigation.

LILRB4 plays a vital role in the progression of various cancers, but the mechanism of LILRB4 remains controversial. Our previous study revealed that LILRB4 supports AML cell infiltration and suppresses T-cell activity via the ApoE/LILRB4/SHP-2/arginase-1 signaling axis [20]. FTO remarkably promoted the expression of LILRB4 by suppressing the YTHDF2-mediated decay of m6A-modified LILRB4 mRNA and maintained LILRB4 mRNA stability [21]. There is no evidence on the function and mechanism of LILRB4 in the development of MM. In our work, we found that IKZF1 could bind to the promoters of LILRB4, transactivate the expression of LILRB4 and promote the development of MM through the IKZF1/LILRB4/STAT3/PFKFB1 signaling axis. Other factors, including epigenetic and transcriptional regulation, could regulate LILRB4 expression and function, but the mechanism and the relationship between these transcription factors need to be further investigated.

In summary, LILRB4 was highly expressed in MM cells and adversely correlated with patient survival. Mechanistically, the IKZF1-LILRB4-STAT3-PFKFB1 pathways are involved in maintaining the proliferation capability and metabolic states of MM cells. Blocking LILRB4 signaling with an anti-LILRB4 monoclonal antibody (C84) efficiently inhibited the proliferation of MM cells. More efforts are needed to delineate the detailed regulatory networks of LILRB4 in MM development, which may benefit the development of new strategies for the treatment of MM.

## Method

### Mice

NOD-SCID mice were purchased from Shanghai SLAC Laboratory Animal Co. Ltd. and bred at the Animal Core Facility. For all experiments, 6- to 8-week-old NOD-SCID mice were used. Animal experiments were evaluated and approved by our institution and performed under the Guideline for Animal Care at Shanghai Jiao Tong University, School of Medicine.

### shRNA construction and blocking assay of LILRB4 in MM cells

To evaluate the function of LILRB4 in MM cells, the lentiviral vector pLKO.1 was used to knock down the expression of human LILRB4. In brief, the pLKO.1 and packaging plasmids of pSPAX2 and pMD2G were cotransfected into 293T cells to produce lentiviruses, which were further used for subsequent infection in human MM cells, including ARP-1 and MM1.S, LP-1 and OPM-2. For the establishment of the xenoengraftment model, 2 × 10^6^ MM cells were injected subcutaneously into nude or NOD-SCID mice, followed by evaluation of the tumor sizes and weight at 16–40 days after transplantation. All mice were sacrificed 4–8 weeks after transplantation.

### Statistical analysis

Statistical analysis was performed using GraphPad and SPSS software, version 19.0. Data are represented as the mean ± SEM. All experiments were performed independently at least 3 times. Data were analyzed with Student’s t test (two-tailed), one-way ANOVA with Tukey’s multiple comparison test, or two-way ANOVA with Sidak’s multiple comparison test according to the experimental design. The overall survival of different groups was compared using the Kaplan‒Meier method with a log-rank test. Sample sizes were chosen according to previously performed power analysis. For the animal experiment, a sample size of 5 mice per group and an experimental replicate was calculated and used. No sample blinding or randomization was performed. Gene expression, CFU assay and proliferation experiments were performed in triplicate (3 technical replicates) at least 3 times (3 independent experiments). Chromatin immunoprecipitation experiments were repeated at least 3 times. No data were excluded from the analysis. Statistical significance was set at p < 0.05 (*P < 0.05; **P < 0.01; ***P < 0.001).

### Supplementary information

Supplement material

Western blot original data

qPCR original data

## Data Availability

The data supporting the findings of this study are available from the corresponding author upon reasonable request.
