# Supplementary material for: LILRB4 regulates multiple myeloma development through STAT3-PFKFB1 pathway
Source: Cell Death Dis. 2024 Jul 18;15(7):515. doi: 10.1038/s41419-024-06883-4 (PMC11258265; doi:10.1038/s41419-024-06883-4)
Supplement: Supplementary file 1 — Supplement material [file 41419_2024_6883_MOESM1_ESM.pdf]

Supplemental materials

Antibodies and Reagents

Antibodies: Anti-human CD85K-APC (eBioscience, CA, USA, #17-5139-42); Anti-AnnexinV-APC (BD Pharmingen, CA, USA, #550475); anti-STAT3 (CST, CA, USA, #30835), anti-phospho-STAT3 (Tyr705) (CST, CA, USA, #9145); anti-IKZF1 (CST, CA, USA, #5543)； anti–β-actin-HRP (MBL, CA, Japan, PM053-7).

Reagents: XF Cell Mito Stress Test Kit (Agilent, China, #103015-100), XF Glycolysis Stress Test Kit (Agilent, #103020-100), ATP Bioluminescence Assay Kit HS II (Roche, China, #11699709001).

Cell lines

The human MM cell lines MM.1S, OPM2, LP-1, ARP1 and RPMI 8226 (ATCC) were cultured in RPMI-1640 medium supplemented with 20% fetal bovine serum (FBS) and 1% penicillin streptomycin. HEK293T cells were cultured in DMEM supplemented with 10% FBS.

Luciferase Reporter Assays

To evaluate the transcriptional regulation of LILRB4 by IKZF1 and PFKFB1 by STAT3, we performed a luciferase reporter assay in 293T cells. In brief, 293T cells were cotransfected with PLVX-STAT3-strepII (or empty vector) with the pGL4.27-PFKFB1 promoter or PLVX-IKZF1-strepII with the pGL4.27-LILRB4 promoter for 24 hrs. Luciferase activities were measured by using a luciferase reporter system (GloMax®Multi Instrument). Measurements were calculated as firefly luciferase units vs. Renilla luciferase units.

Chromatin immunoprecipitation (ChIP) assay

The ChIP assay was performed using the ChIP Assay Kit (Beyotime). 293T cells were cotransfected with the PLVX-STAT3-strepII plasmid with the pGL4.27-PFKFB1-promoter or PLVX-IKZF1-HA plasmid pGL4.27-LILRB4-promoter plasmid and crosslinked with 1% formaldehyde (Sigma–Aldrich) at 37 °C for 10 min, followed by incubation with anti-strepII or HA antibodies (Sigma) or control rabbit IgG (Santa Cruz) at 4 °C overnight before immunoprecipitation. For the sample input, 1% of the sonicated precleared DNA was purified at the same time as the precipitated immune complex. The ChIP samples were purified by the Gel and PCR Clean-up Kit (Nucleospin). The STAT3- or LILRB4-binding sequence was amplified by semiquantitative PCR using primers specific for human PFKFB1 and LILRB4, as listed in Table S1.

Immunoblot analysis

For immunoblot analysis, whole cell lysates were electrophoresed onto 10% sodium dodecyl sulfate–polyacrylamide gels and transferred to nitrocellulose membranes (Millipore). After blocking with a 5% solution of nonfat dried milk, the membranes were incubated with the following primary antibodies at 4 °C overnight: anti-phospho-STAT3 (Tyr705) (CST, CA, USA, #9145), anti-STAT3 (CST, CA, USA, #30835) and anti-β-actin (MBL). The membranes were further incubated with the appropriate horseradish peroxidase-conjugated secondary antibody (Sigma) at room temperature for 1 hr, followed

by the detection of the protein level.

Quantitative RT–PCR

LILRB4-knockdown MM cells and scrambled MM cells were FACS-purified for RNA extraction. First-strand cDNA was reverse transcribed using AMV reverse transcriptase (TaKaRa). PCRs were performed according to the manufacturer's instructions (Roche). The experiments were conducted in triplicate with the Applied Biosystems 7900HT, and the mRNA levels were normalized to the level of β-actin RNA transcripts. The sequences of primers used are listed in Table S1.

Metabolic analyses of the oxygen consumption rate, extracellular acidification rate and ATP level

The oxygen consumption rate (OCR) and extracellular acidification rate (ECAR) were detected in LILRB4-knockdown and scrambled ARP-1 cells with an XF Cell Mito Stress Test Kit (Seahorse, 103015-100) and an XF Glycolysis Stress Test Kit (Seahorse, 103020-100) using a Seahorse XF96 analyzer according to the manufacturer's instructions. For the detection of OCR, 1.5 μM oligomycin, 2 μM FCCP and 0.5 μM rotenone/antimycin were loaded into injection ports A, B and C, respectively. During sensor calibration, the indicated cells were incubated in 175 μL of assay medium (XF Base Medium supplemented with 2 mM glutamine, 1 mM pyruvate, and 10 mM glucose, pH 7.4 at 37 °C) in a 37 °C CO_2-free incubator. For the ECAR test, injection port A on the sensor cartridge was loaded with 10 mM glucose. Then, 2.5 μM oligomycin was loaded into port B, and 100 mM 2-DG was loaded into port C. During sensor calibration, the cells were incubated in 175 μL of assay medium (XF Base Medium supplemented with 1 mM glutamine alone, pH 7.4 at 37 °C) in a 37 °C CO_2-free incubator.

Lactate measurement

Lactate levels in the supernatant were measured according to the manufacturer's protocol (Sigma–Aldrich, MAK064). Briefly, 6 $X10^5$ LILRB4-knockdown and scrambled ARP-1 cells were plated in RPMI-1640 medium and supplemented with 20% fetal bovine serum (FBS), followed by incubation at 37 °C for 4 hr. The supernatant was centrifuged at 12,000 g at 4 °C for 5 min to remove cell debris before the analysis of lactate levels. Lactate levels were determined by a spectrophotometer according to the manufacturer's protocol.

Figure S1

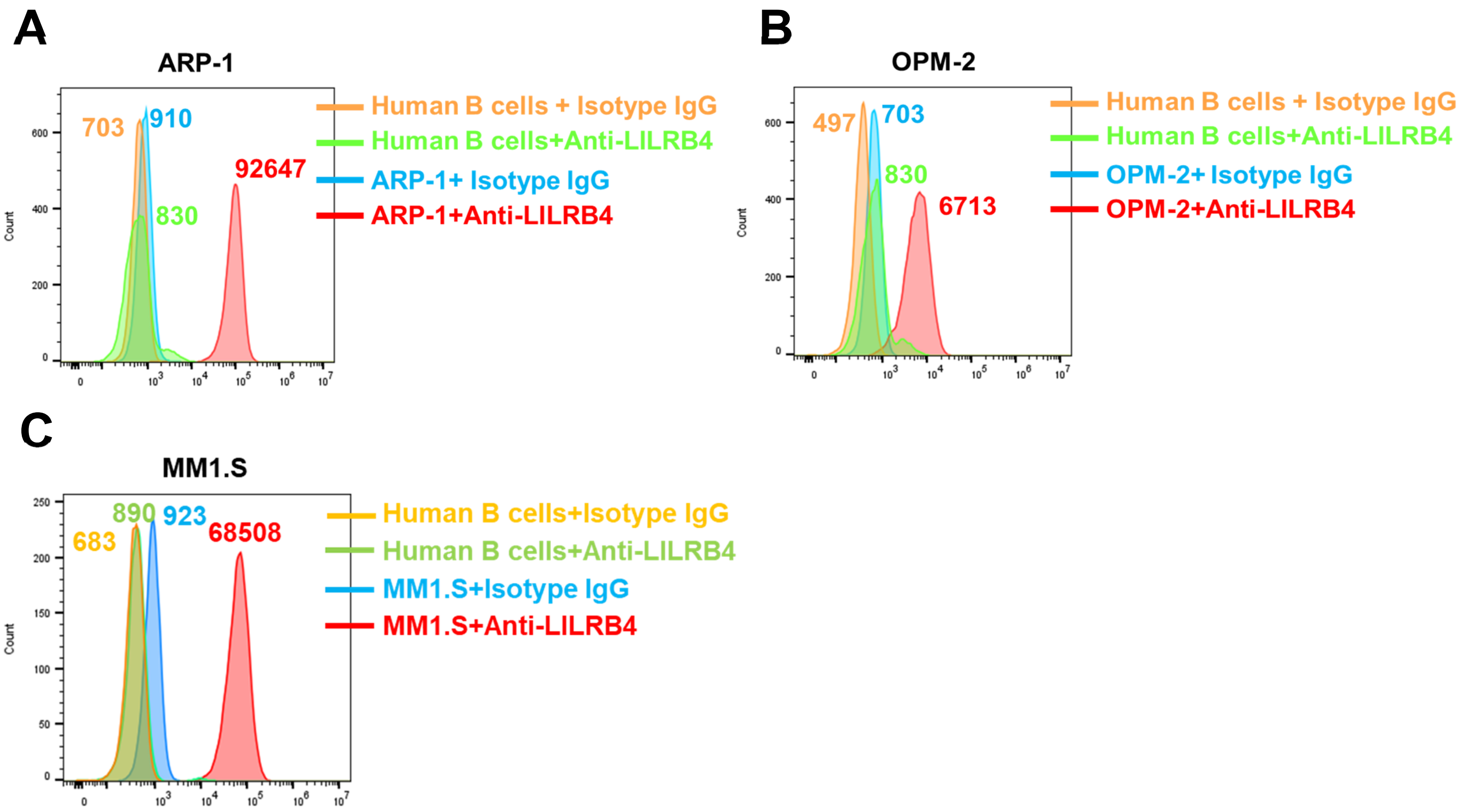
A
ARP-1
703
910
830
92647
Human B cells + Isotype IgG
Human B cells+Anti-LILRB4
ARP-1+ Isotype IgG
ARP-1+Anti-LILRB4
Count
B
OPM-2
497
703
830
6713
Human B cells + Isotype IgG
Human B cells+Anti-LILRB4
OPM-2+ Isotype IgG
OPM-2+Anti-LILRB4
Count
C
MM1.S
683
890
923
68508
Human B cells+Isotype IgG
Human B cells+Anti-LILRB4
MM1.S+Isotype IgG
MM1.S+Anti-LILRB4
Count

Figure S1, related to Figure 1. LILRB4 is highly expressed on multiple myeloma cells. (A-C) LILRB4 expression levels were quantified by flow cytometric analysis in ARP-1 (A), OPM2 (B), MM1.S (C) and normal B cells.

Figure S2

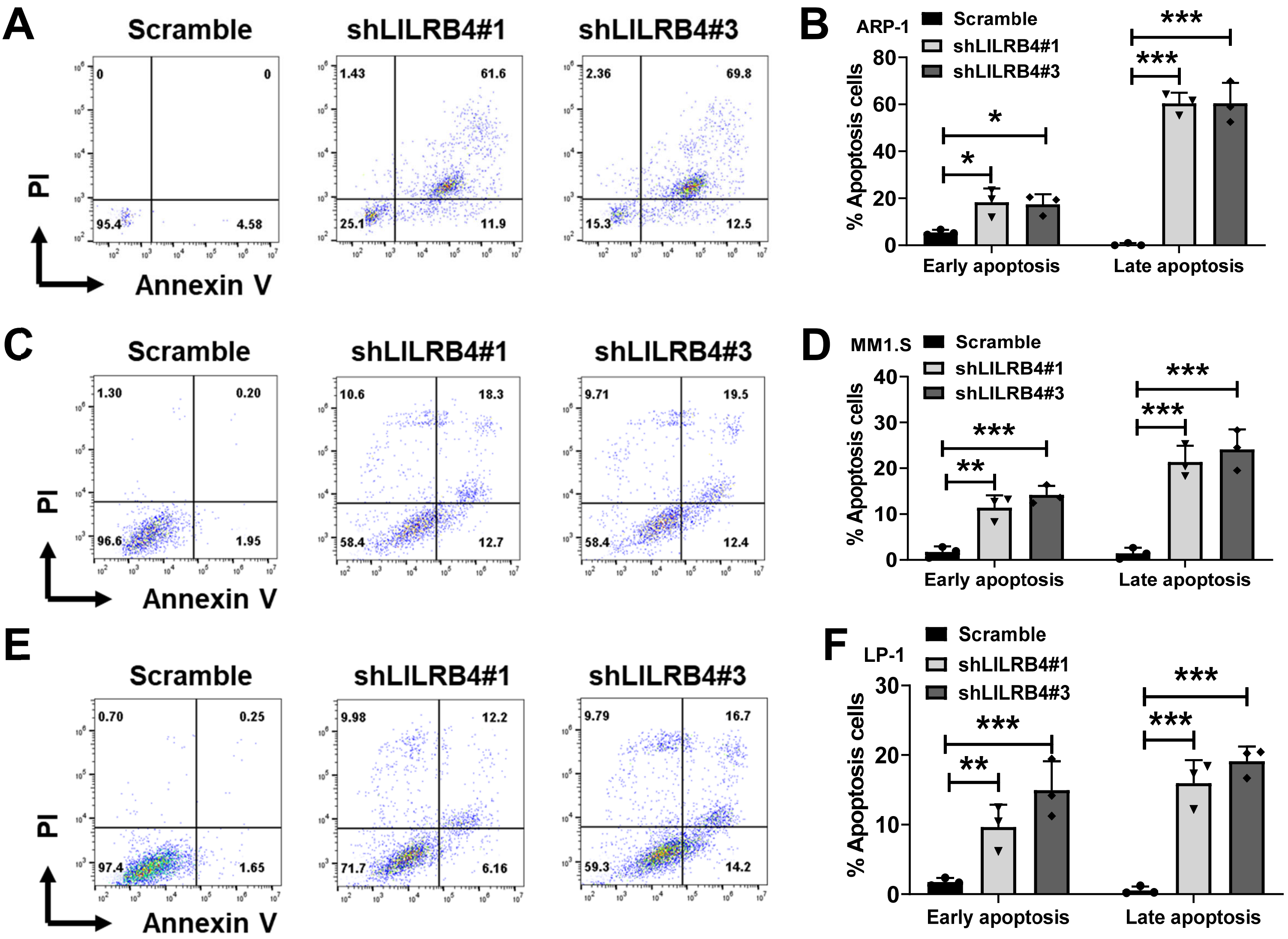

A
Scramble
shLILRB4#1
shLILRB4#3
0
0
95.4
4.58
1.43
61.6
25.1
11.9
2.36
69.8
15.3
12.5
PI
Annexin V
B
ARP-1
Scramble
shLILRB4#1
shLILRB4#3
% Apoptosis cells
Early apoptosis
Late apoptosis
C
Scramble
shLILRB4#1
shLILRB4#3
1.30
0.20
96.6
1.95
10.6
18.3
58.4
12.7
9.71
19.5
58.4
12.4
PI
Annexin V
D
MM1.S
Scramble
shLILRB4#1
shLILRB4#3
% Apoptosis cells
Early apoptosis
Late apoptosis
E
Scramble
shLILRB4#1
shLILRB4#3
0.70
0.25
97.4
1.65
9.98
12.2
71.7
6.16
9.79
16.7
59.3
14.2
PI
Annexin V
F
LP-1
Scramble
shLILRB4#1
shLILRB4#3
% Apoptosis cells
Early apoptosis
Late apoptosis

Figure S2, related to Figure 2. LILRB4 supports MM cell proliferation in vitro. (A-B) Representative flow cytometric analyses of percentages of early and late apoptotic cells in scrambled or LILRB4-knockdown (shLILRB4#1 and #3) ARP-1 cells. (C-D) Representative flow cytometric analyses of percentages of early and late apoptotic cells in scrambled or LILRB4-knockdown (shLILRB4#1 and #3) MM1.S cells. (E-F) Representative flow cytometric analyses of percentages of early and late apoptotic cells in scrambled or LILRB4-knockdown (shLILRB4#1 and #3) LP-1 cells. Two-way ANOVA with Sidak's multiple comparison test (B, D and F) was used for the comparison of statistical significance. *, $p<0.05$; **, $p<0.01$; ***, $p<0.001$.

Table S1. Primer sequences

Human primers for qRT-PCR	Sequences
STAB1-F	AACCACGTTTGTCACTCATGT
STAB1-R	CGGCAGTCCTGGGTTATCTG
PTGS-F	CTGGCGCTCAGCCATACAG
PTGS -R	CGCACTTATACTGGTCAAATCCC
EGR1-F	GGTCAGTGGCCTAGTGAGC
EGR1-R	GTGCCGCTGAGTAAATGGGA
CDKN1A-F	TGTCCGTCAGAACCCATGC
CDKN1A -R	AAAGTCGAAGTTCCATCGCTC
ALDH7A1-F	CCAGTATGCGTGGCTGAAAGA
ALDH7A1-R	CAGGGCAATAGGTCGTAATAACC
PSPH-F	GAGGACGCGGTGTCAGAAAT
PSPH -R	GGTTGCTCTGCTATGAGTCTCT
GADD45A-F	GAGAGCAGAAGACCGAAAGGA
GADD45A -R	CACAACACCACGTTATCGGG
BCL2A1-F	TACAGGCTGGCTCAGGACTAT
BCL2A1-R	CGCAACATTTTGTAGCACTCTG
FLT1-F	TTTGCCTGAAATGGTGAGTAAGG
FLT1-R	TGGTTTGCTTGAGCTGTGTTC
SAGE1-F	TACCAGGGATCTGCATTCTACC
SAGE1-R	CTGTGGGACCAGTTGACAAGA
IKZF1-F	CATCAGCCCGATGTACCAGC
IKZF1-R	CCTCGTTGTTGCTCTCGGT
IKZF3-F	GCTCATACAGACCCGCATGAT
IKZF3-R	AACTGGAACCATCTCCGAGGT
PFKFB1-F	TACCAGAGAACGACGGTCACT
PFKFB1-R	CTGCAATTATGCCAGGGTCATTA
β-ACTIN-F	AGAGCTACGAGCTGCCTGAC
β-ACTIN-R	AGCACTGTGTTGGCGTACAG
**Primers for shRNAs**	**Target sequences**
Human shLILRB4#1	GCTCATAGTCTCAGGATCCTT
Human shLILRB4#3	CTCGGGAGTACCGTCTGGATA
**Primers for vector**	**Sequences**
Human PFKFB1-F	TCTATTTCCGGTGAATTCCTCGAGATGTCTCCAG AGATGGGAGA
Human PFKFB1-R	GCACCTCCAGGGATCCGCGGCCGCaGTAGTGGG CTGGGACAGTAT
Human IKZF1-F	TCTATTTCCGGTGAATTCCTCGAGATGGAAGATA TACAAACAAA
Human IKZF1-R	GCACCTCCAGGGATCCGCGGCCGCaCTTCAGCA GGGCTCTGTGTT
Human ALDH7A1-F	TCTATTTCCGGTGAATTCCTCGAGATGTGGCGCC

Human ALDH7A1-R	TTCCTCGCGC GCACCTCCAGGGATCCGCGGCCGCaCTGAAACT TGATTCCTTGGG
**Primers for ChIP**	**Sequences**
PFKFB1-promoter-ChIP-3-F	CTTGGCATGAGGAACTTTAG
PFKFB1-promoter-ChIP-3-R	TGGGCCCAAGTAGAATGTCA
PFKFB1-promoter-ChIP-4-F	TGACATTCTACTTGGGCCCA
PFKFB1-promoter-ChIP-4-R	CTTAGGAGTCGCACCGAATG
LILRB4-promoter-ChIP-4-F	AGGAGGACACGGCTCTGATA
LILRB4-promoter-ChIP-4-R	GGCGTCTCCTCCCAGGGGCC
