# Supplementary material for: LILRB4 regulates multiple myeloma development through STAT3-PFKFB1 pathway
Source: Cell Death Dis. 2024 Jul 18;15(7):515. doi: 10.1038/s41419-024-06883-4 (PMC11258265; doi:10.1038/s41419-024-06883-4)
Supplement: Supplementary file 2 — Western blot original data [file 41419_2024_6883_MOESM2_ESM.pptx]

## Slide 1
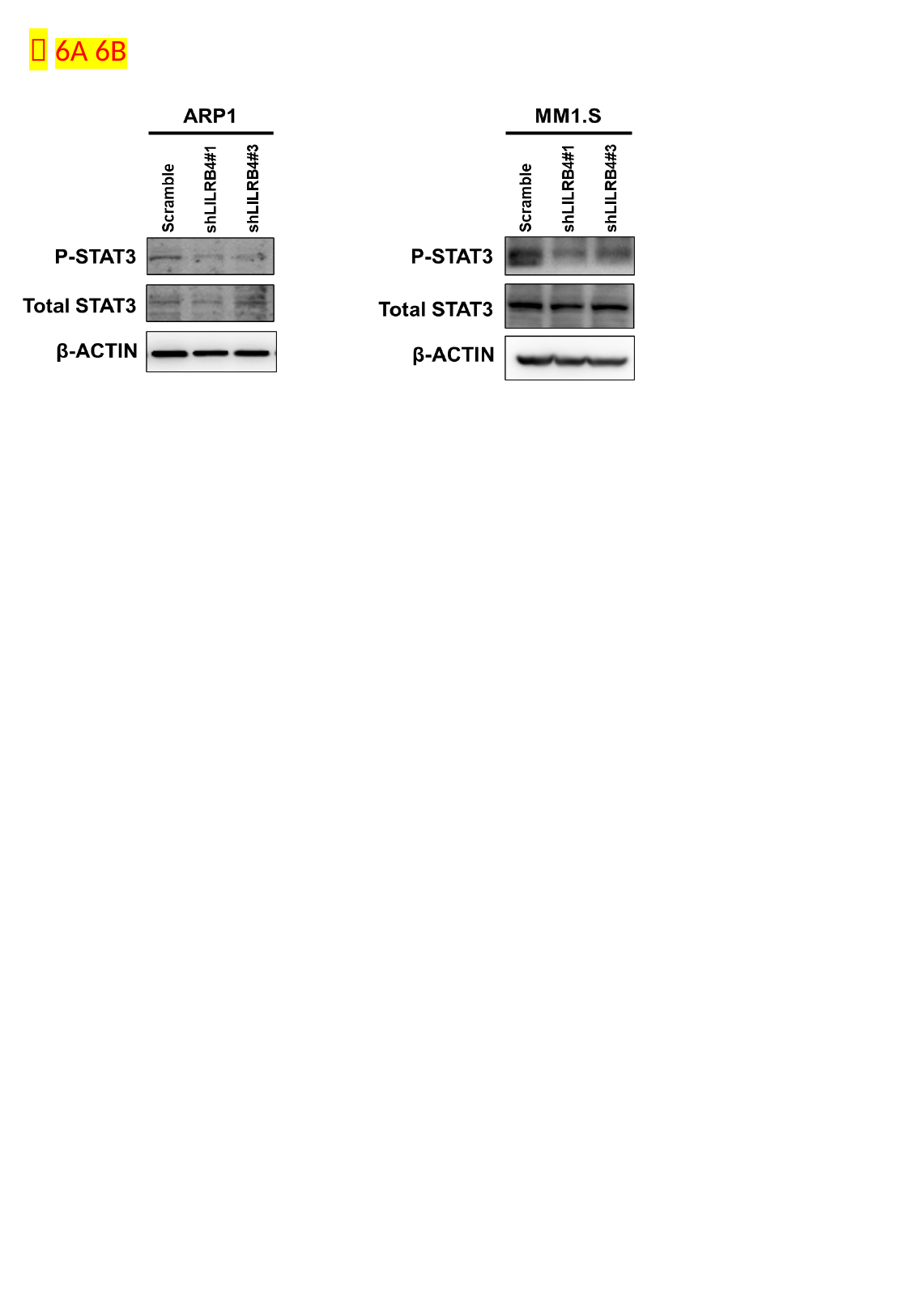

缺6A 6B

## Slide 2
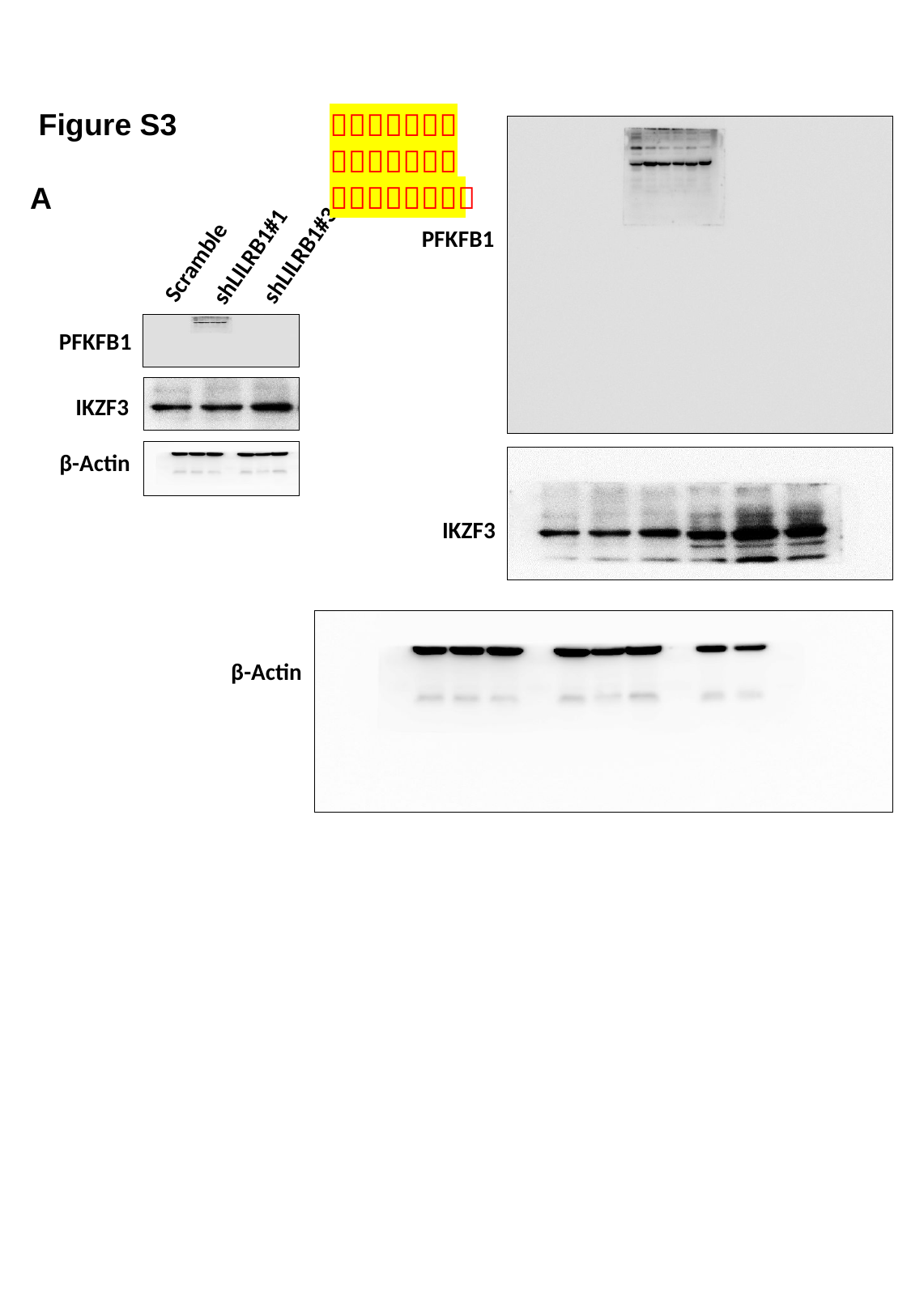

Figure S3
原来的图，内参对不上，更换能对的上的新图吧？
PFKFB1
IKZF3
A
 shLILRB1#3
 shLILRB1#1
 Scramble
PFKFB1
β-Actin
IKZF3
β-Actin

## Slide 3
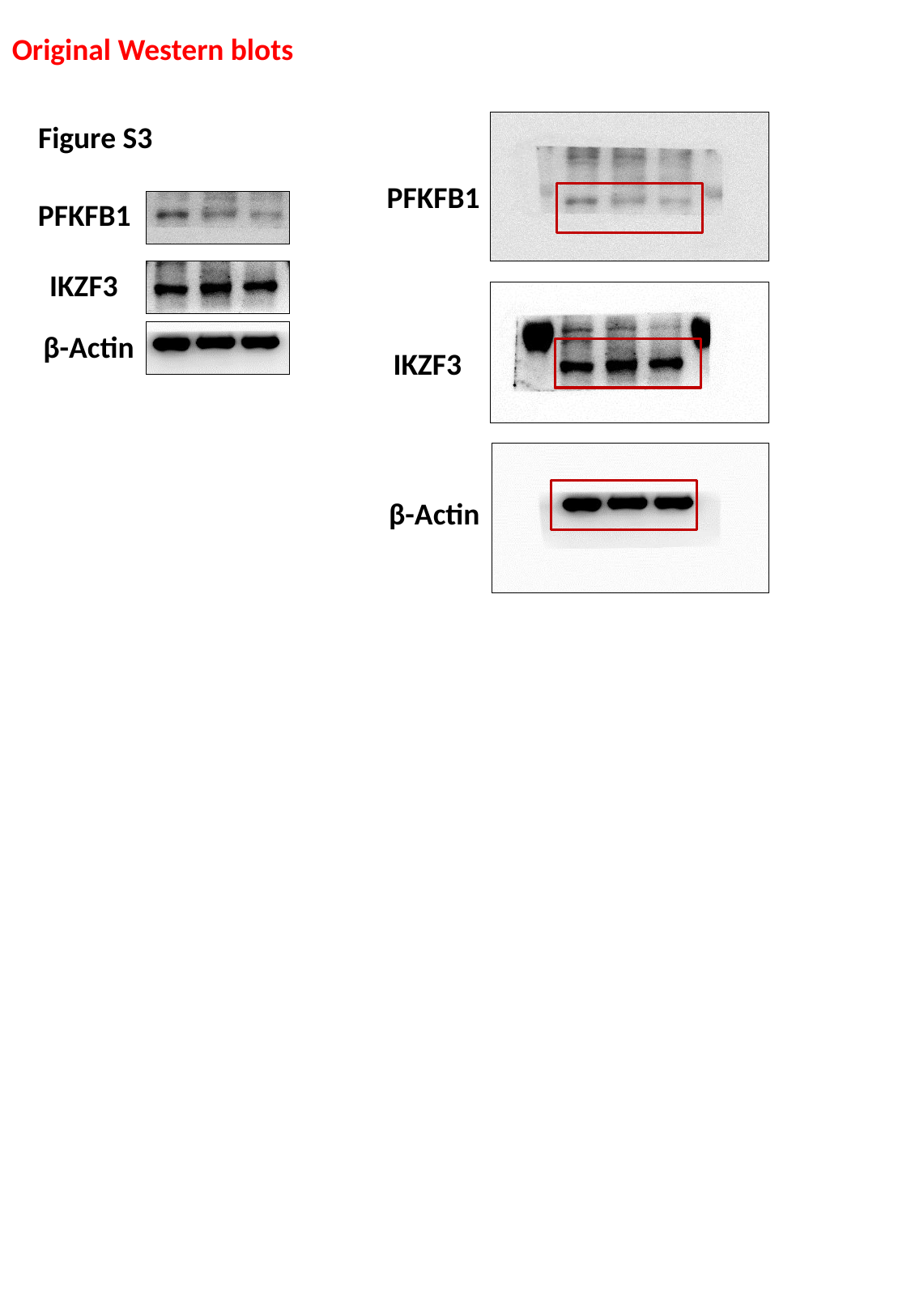

Original Western blots
Figure S3
PFKFB1
PFKFB1
IKZF3
β-Actin
IKZF3
β-Actin

## Slide 4
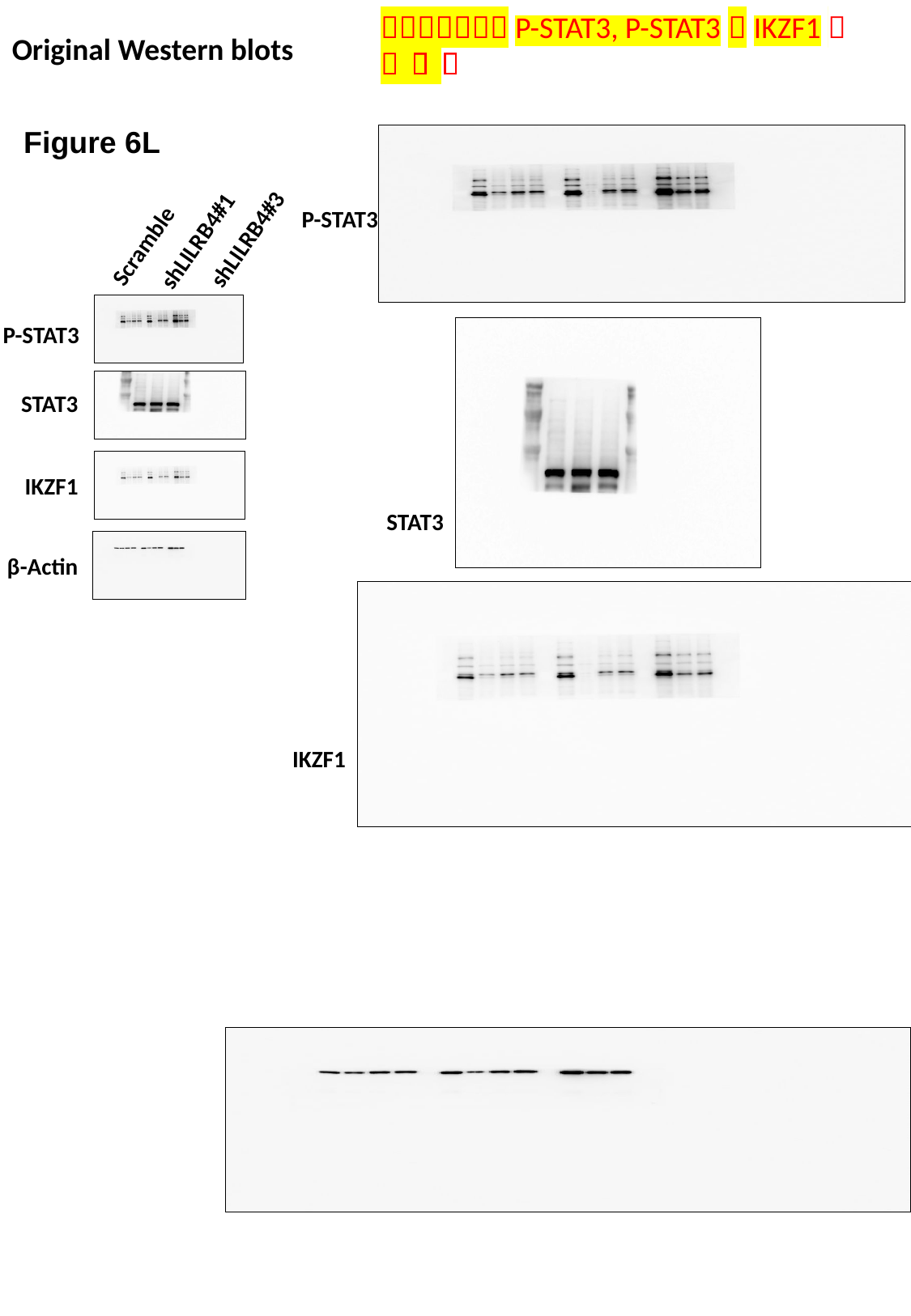

这张图我没杂过P-STAT3, P-STAT3和IKZF1是一张图，只是深浅不一样，换新的吧
Original Western blots
Figure 6L
 Scramble
IKZF1
β-Actin
 shLILRB4#3
 shLILRB4#1
P-STAT3
STAT3
P-STAT3
STAT3
IKZF1

## Slide 5
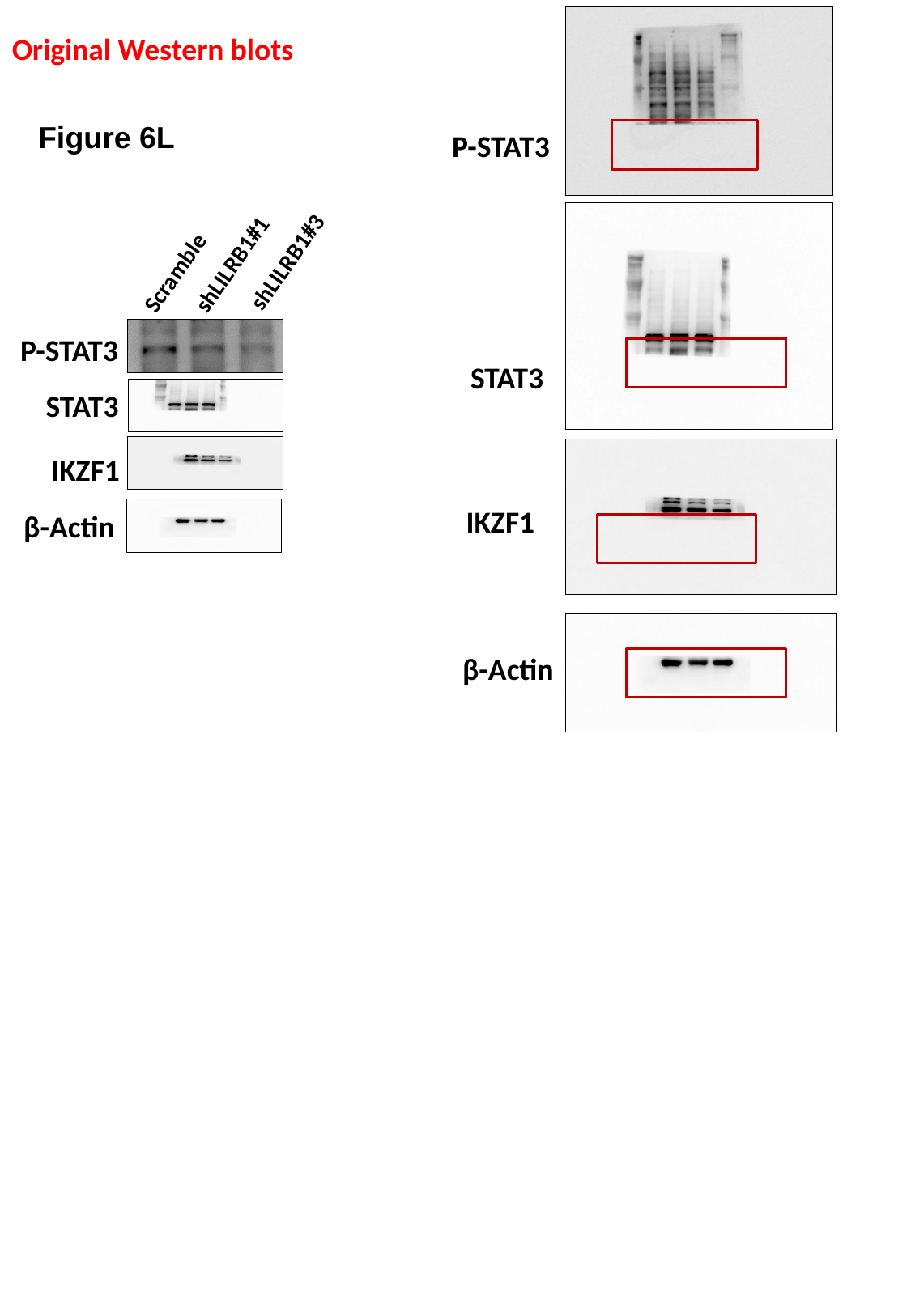

P-STAT3
Original Western blots
Figure 6L
 shLILRB1#3
 shLILRB1#1
 Scramble
P-STAT3
STAT3
IKZF1
β-Actin
STAT3
IKZF1
β-Actin

## Slide 6
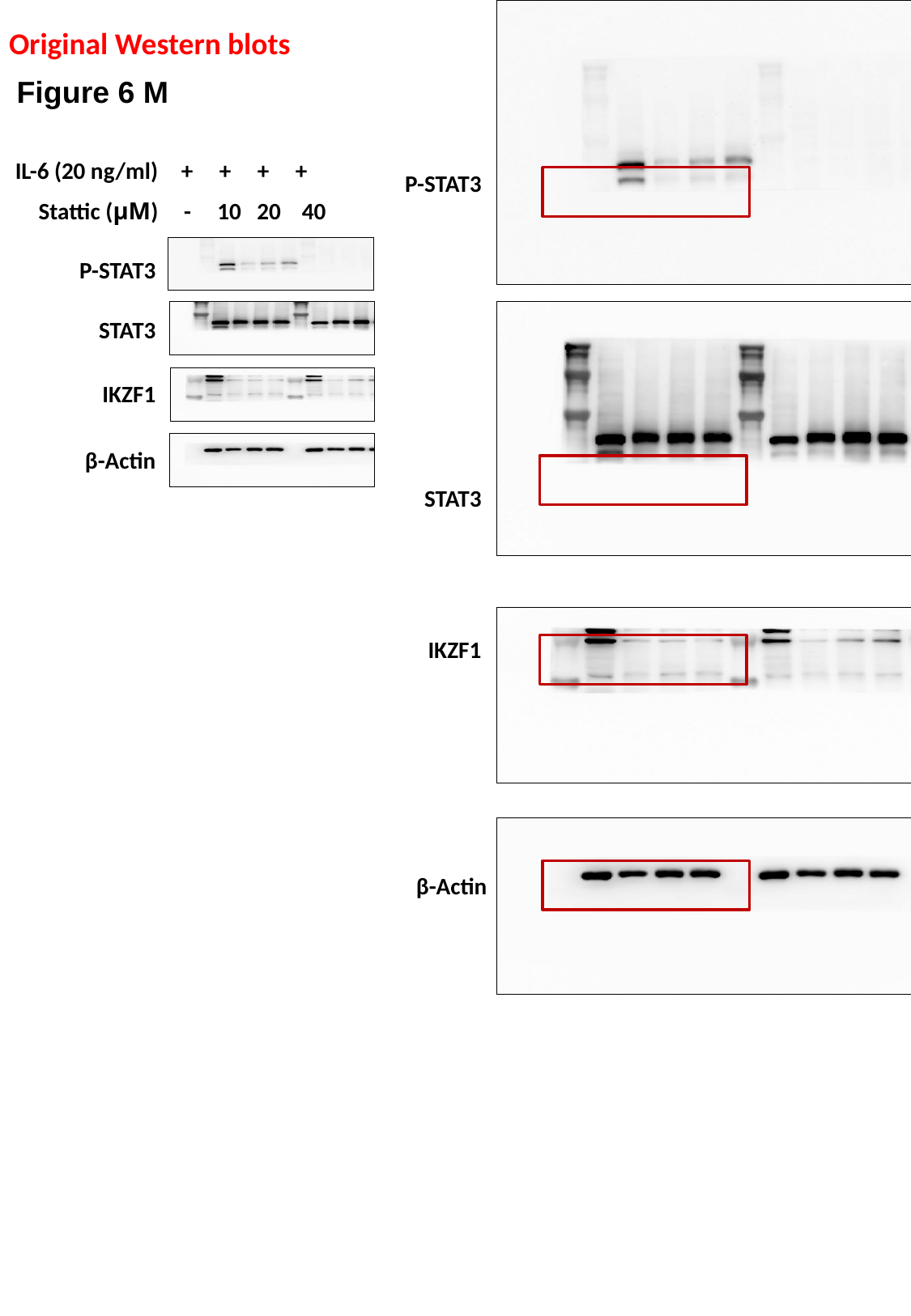

P-STAT3
STAT3
IKZF1
β-Actin
Original Western blots
Figure 6 M
 + + + +
IL-6 (20 ng/ml)
 - 10 20 40
Stattic (μM)
IKZF1
β-Actin
P-STAT3
STAT3

## Slide 7
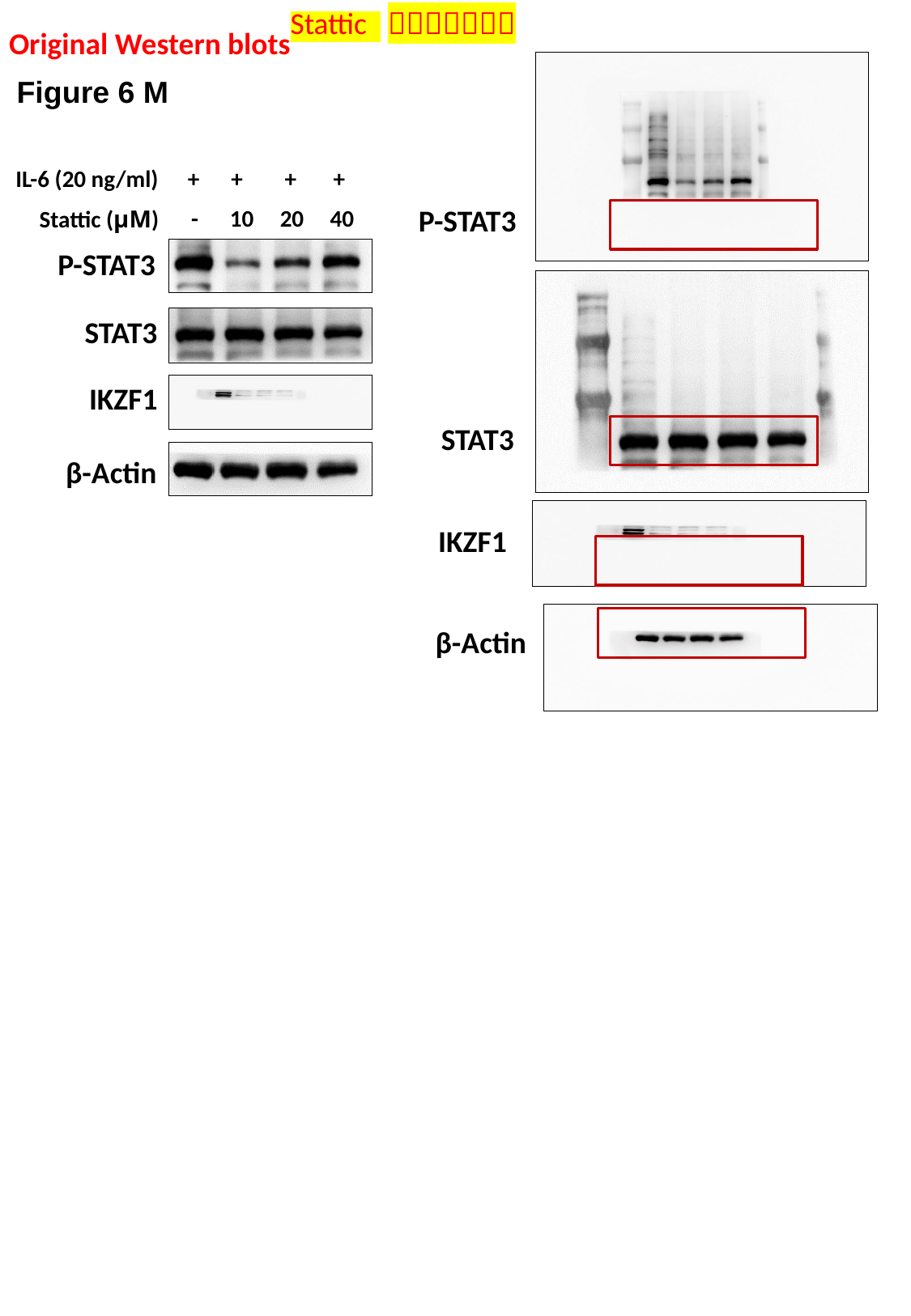

Stattic 两图都可以使用
Original Western blots
P-STAT3
STAT3
IKZF1
β-Actin
Figure 6 M
 + + + +
IL-6 (20 ng/ml)
 - 10 20 40
Stattic (μM)
P-STAT3
STAT3
IKZF1
β-Actin

## Slide 8
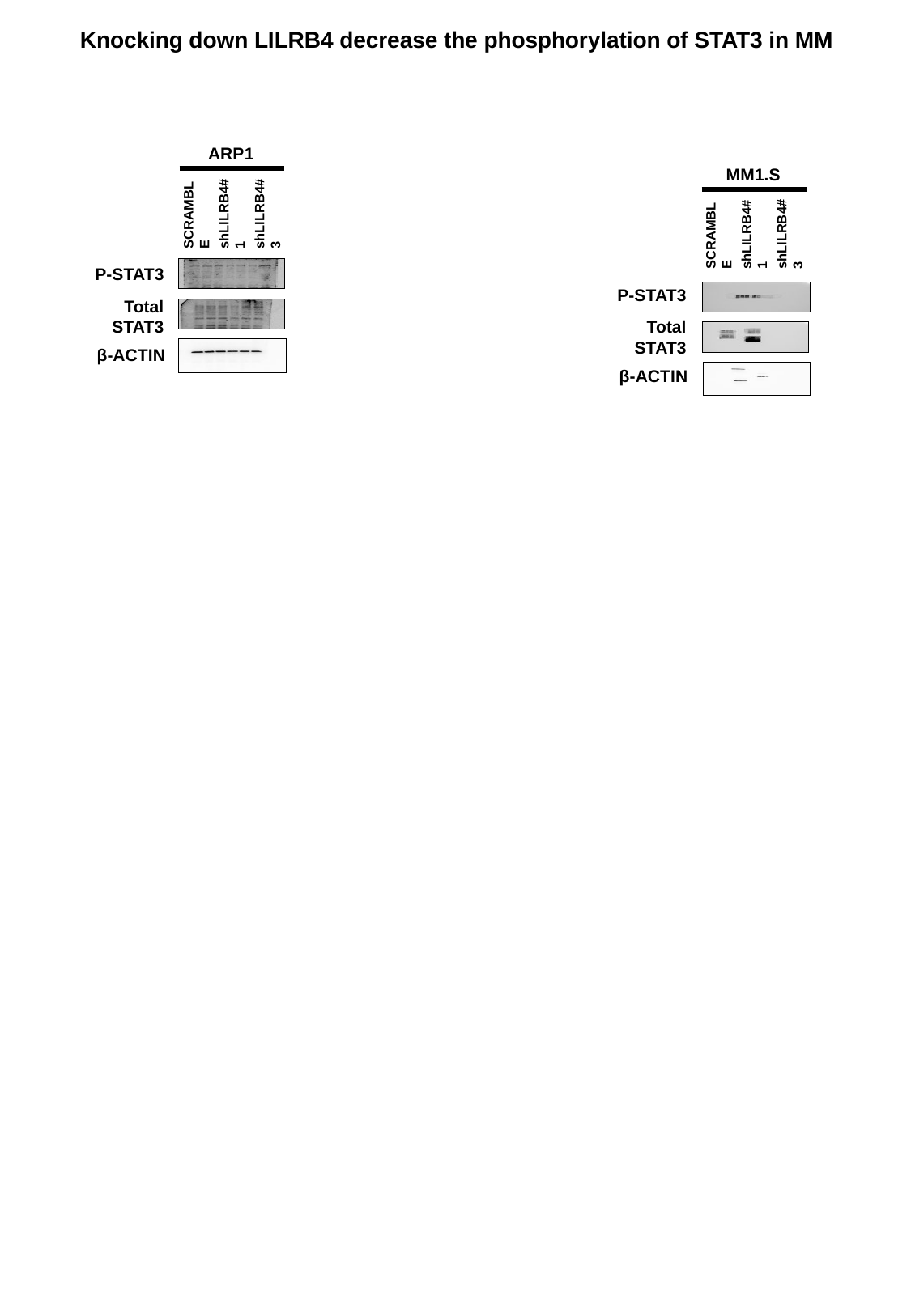

Knocking down LILRB4 decrease the phosphorylation of STAT3 in MM
ARP1
shLILRB4#3
SCRAMBLE
shLILRB4#1
P-STAT3
Total STAT3
β-ACTIN
MM1.S
shLILRB4#3
SCRAMBLE
shLILRB4#1
P-STAT3
Total STAT3
β-ACTIN
